# Endoplasmic Reticulum Stress Impairs Insulin Receptor Signaling in the Brains of Obese Rats

**DOI:** 10.1371/journal.pone.0126384

**Published:** 2015-05-15

**Authors:** Lina Liang, Jing Chen, Libin Zhan, Xiaoguang Lu, Xiaoxin Sun, Hua Sui, Luping Zheng, Hong Xiang, Fuliang Zhang

**Affiliations:** 1 College (Institute) of Integrative Medicine, Dalian Medical University, Dalian, Liaoning, China; 2 Department of Traditional Chinese Medicine, the Second Affiliated Hospital, Dalian Medical University, Dalian, Liaoning, China; 3 Department of Emergency Medicine, Zhongshan Hospital, Dalian University, Dalian, Liaoning, China; 4 Institute of Public Hygiene, Dalian Medical University, Dalian, Liaoning, China; Universidade de São Paulo, BRAZIL

## Abstract

The incidence of obesity is increasing worldwide. It was reported that endoplasmic reticulum stress (ERS) could inhibit insulin receptor signaling by activating c-Jun N-terminal kinase (JNK) in the liver. However, the relationship between ERS and insulin receptor signaling in the brain during obesity remains unclear. The aim of the current study was to assess whether ERS alters insulin receptor signaling through the hyper-activation of JNK in the hippocampus and frontal cortex in the brains of obese rats. Obesity was induced using a high fat diet (HFD). The Morris water maze test was then performed to evaluate decreases in cognitive function, and western blot was used to verify whether abnormal insulin receptor signaling was induced by ERS in HFD rats exhibiting cognitive decline. In addition, to determine whether ERS activated JNK and consequently impaired insulin receptor signaling, SH-SY5Y cells were treated with the JNK inhibitor, SP600125, followed by tunicamycin or thapsigargin, and primary rat hippocampal and cortical neurons were transfected with siRNA against IRE1α and JNK. We found that the expression of phosphorylation of PKR-like kinase (PERK), phosphorylation of α subunit of translation initiation factor 2 (eIF2α), and phosphorylation of inositol-requiring kinase-1α (IRE-1α) were increased in the brains of rats with HFD when compared with control rats. The level of serine phosphorylation of insulin receptor substrate-1 (IRS-1) was also increased, while protein kinase B (PKB/Akt) was reduced. ERS was also found to inhibit insulin receptor signaling via the activation of JNK in SH-SY5Y cells, primary rat hippocampal, and cortical neurons. These results indicate that ERS was increased, thereby resulting in impaired insulin receptor signaling in the hippocampus and frontal cortex of obese rats.

## Introduction

The incidence of obesity is increasing worldwide, in part due to changes in nutritional habits and lifestyle. The central nervous system (CNS) is one of the systems affected by obesity. Compelling evidence now suggests a link between obesity and dementia [1]. Several studies have reported that obesity is associated with cognitive impairment [2,3,4] and longitudinal studies have revealed that obesity could accelerate cognitive decline [5,6]. Although an increasing number of studies have focused on cognitive impairment induced by obesity, its underlying mechanisms remain unclear.

Insulin receptors are expressed ubiquitously in the brain [7]. Circulating insulin can enter the brain by crossing the blood—brain barrier, where it can activate signaling pathways in the CNS. Metabolic and cognitive disorders such as obesity, type 2 diabetes mellitus (T2DM), and Alzheimer’s disease (AD) are associated with insulin resistance within the CNS, which may result from genetic polymorphisms or long-term exposure to elevated levels of circulating insulin due to peripheral insulin resistance.

The endoplasmic reticulum (ER) comprises a sophisticated luminal network that is the primary site of protein synthesis, maturation, folding, and transport [8,9]. Perturbation of these processes during different pathological states results in a condition known as ER stress (ERS), which leads to the activation of a complex signaling network termed the unfolded protein response (UPR) [10]. It was reported that ERS could induce peripheral insulin resistance by regulating oxidative stress [11,12] and leptin resistance [13] in the hypothalamus. ERS can also inhibit insulin receptor signaling by activating JNK in the liver [14]. However, the relationship between ERS and insulin receptor signaling in the CNS, particularly in the hippocampus and frontal cortex which are responsible for learning and memory of obese rats, remains unclear.

In the present study, we hypothesized that ERS induces insulin resistance in the brains of obese rats. Defining the relationship between ERS and insulin receptor signaling in the brains of obese rats during cognitive decline will help determine the mechanism of metabolic and cognitive impairment, and may facilitate the development of novel therapeutic agents in diabetes-associated cognitive decline (DACD).

## Materials and Methods

### Ethics statement

All animal experiments were conducted in accordance with the NIH Principles of Laboratory Animal Care and the institutional guidelines for the care and use of laboratory animals at Dalian Medical University. All experiments were approved by the Committee on the Ethics of Animal Experiments of Dalian Medical University (Permit Number: SYXK (Liao) 2009–0102). We attest that every effort was made to minimize the number of animals used and their suffering.

### Animals

Adult male Sprague-Dawley rats (6 weeks of age, 180–220 g) were obtained from the Experimental Animal Center, Dalian Medical University, Dalian, China. Rats were housed in a 12 h light-dark cycle and were permitted free access to food and water. Animals were assigned to control and high fat diet (HFD) groups, and were fed a standard or high fat diet (40% energy from fat, Anlimo Technology, Nanjing, China) for 6 weeks, respectively.

### Morris water maze test

The maze apparatus consisted of a round stainless steel water tank (200 cm in diameter, 50 cm in height and filled to a depth of 30 cm with water maintained at 26±1°C), a submerged transparent platform (9 cm in diameter, 29 cm in height and located 30 cm from the edge of the tank), and an automatic photographic recording and analysis system (Institute of Materia Medica, Chinese Academy of Medical Sciences, Beijing, China). Black and white spatial cues located on the walls of the room provided the rats with orientation. On the first day, rats were permitted to swim freely in the tank for 120 s without the platform to adapt to the new conditions. Over the following 4 days, rats were trained with 4 trials per day at intervals of 60 s. The platform location was submerged 2 cm below the water surface in a fixed location, and the starting point was changed for each trial. Rats were required to swim until they reached the platform. If the rat failed to reach the platform within 120 s, it was gently guided to the platform and allowed to rest there for 60 s. The time taken to reach the platform was measured automatically. On the day after the last acquisition training session, animals were tested in a single 120 s session (probe trial) without the platform. The time to reach the platform (escape latency), and the number of times the animal crossed the site of the original platform were recorded using an automatic photographic recording and analysis system (Institute of Materia Medica, Chinese Academy of Medical Sciences, Beijing, China).

### Cell culture

Euthanized an approximately E18 days pregnant Sprague-Dawley rat by CO_2_. Primary culture of hippocampal and cortical neurons was isolated and cultured as previously described [15]. Briefly, hippocampus and frontal cortex were removed from embryos at 18 days, stripped of meninges and blood vessels and minced. Tissues were dissociated by 0.25% trypsin digestion for 15 min at 37°C and gentle trituration. The dissociated cells were plated at 1.5 × 10^6^ cells/ml on poly-L-lysine-coated 35-mm Petri dishes in Dulbecco’s minimum essential medium (DMEM, Gibco) containing 10% fetal bovine serum (Gibco) and 10% F12 (Gibco). 24h following plating, half of the culture medium was replaced with maintenance medium consisting of Neurobasal medium containing 25mM D-Glucose (GIBCO, USA) supplemented with 2% B27 (GIBCO, USA), 1% Glutamax (GIBCO, USA) and 1 μM insulin. This medium was replaced twice a week. After 72h in vitro, 5 μM cytosine arabinoside was added to the cultures to block proliferation of glial cells. At 9 days in vitro, IRE1α siRNA (Invitrogen, USA), JNK siRNA (Invitrogen, USA) and its control siRNA (Invitrogen, USA) were transfected into rat hippocampal and cortical neurons for 72h using Lipofectamine 2000 Reagent (Invitrogen) according to the manufacturer’s instructions.

SH-SY5Y cells were passaged and maintained in DMEM/F12 medium containing 17.5mM D-Glucose (GIBCO, USA) supplemented with 15% fetal bovine serum (GIBCO, USA), 1 μM insulin, 100 units/ml penicillin G, and 0.1 mg/ml streptomycin (GIBCO, USA), and were cultured at 37°C in a humidified atmosphere containing 5% CO_2_. SH-SY5Y cells were purchased from Shanghai Institutes for Biological Sciences, the Chinese Academy of Sciences (#TCHU 97).

### Sample preparation

For western blot, the animals were anesthetized and decapitated. The frontal cortex and hippocampus were rapidly dissected on ice. All samples were immediately frozen in liquid nitrogen and stored at -80°C until required. The brain samples were homogenized in ice-cold lysis buffer (0.125 M Tris HCl pH 6.8, 0.2 M DTT, 4% SDS, 20% glycerol). The lysates were sonicated for 10 min and centrifuged at 15,000 × g for 5 min to remove insoluble debris. Protein concentrations in the supernatants were determined using a Minim Spectrophotometer (NanoVue Plus, GE Healthcare, Amersham Place, Little Chalfont, Buckinghamshire, HP79NA, UK).

### Western blot

Protein (50 μg per sample) was separated on 8–10% Tris-glycine polyacrylamide gels, and transferred to nitrocellulose membranes. Immunoblots were blocked for 2 h in Tris-buffered saline Tween-20 (TBST: 20 mM Tris-HCl, 150 mM NaCl, pH 7.5, 0.05% Tween 20) containing 5% non-fat milk. The blots were then incubated with primary antibodies in TBST at 4°C overnight. Membranes were washed three times with TBST and subsequently incubated with secondary antibodies for 2 h at 25°C. Membranes were washed again, and developed using Enhanced Chemiluminescence (ECL). The primary antibodies used from Cell Signaling Technologies (Danvers, MA, USA) were: pThr^980^PERK (#3179, 1:800), pSer^51^eIF2α (#3597, 1:800), eIF2α (#9722, 1:800), pThr^183^/Tyr^185^JNK (#9251, 1:800), JNK (#9252, 1:800), pSer^473^PKB/Akt (#4058, 1:1000), PKB/Akt (#9272, 1:1000). Primary antibodies directed against pSer^307^IRS-1 (#GF1005, 1:1000) (Millipore Corporation, Billerica, MA, USA), IRS-1 (#06–248, 1:800) (Millipore Corporation, Billerica, MA, USA), pSer^731^IRS-2 (#ab3690, 1:600) (Abcam plc, Cambridge, UK), pSer^724^IRE1α (#48187, 1:800) (Abcam plc, Cambridge, UK), and β-actin (A2228, 1: 2000) (Sigma-Aldrich, St Louis, MO, USA) were also used. Goat anti-rabbit (#9340, 1:2000) or anti-mouse IgG (#9310, 1:2000) (GE Healthcare, Buckinghamshire, UK) were used as secondary antibodies.

### Statistical analysis

All values are expressed as mean ± standard deviation (SD). Statistical significance was analyzed using Student’s *t*-test and ANOVA, followed by the least-significant difference post hoc using SPSS 13.0 (SPSS, Chicago, IL, USA). Data from the Morris water maze test was analyzed using a repeated-measures ANOVA for comparisons among trials, while an unpaired Student’s *t*-test was used for comparisons between both groups in a given block and for comparison with other results. A value of *p* < 0.05 was considered to be statistically significant.

## Results

### Cognitive performance in the Morris water maze

**Fig 1:**
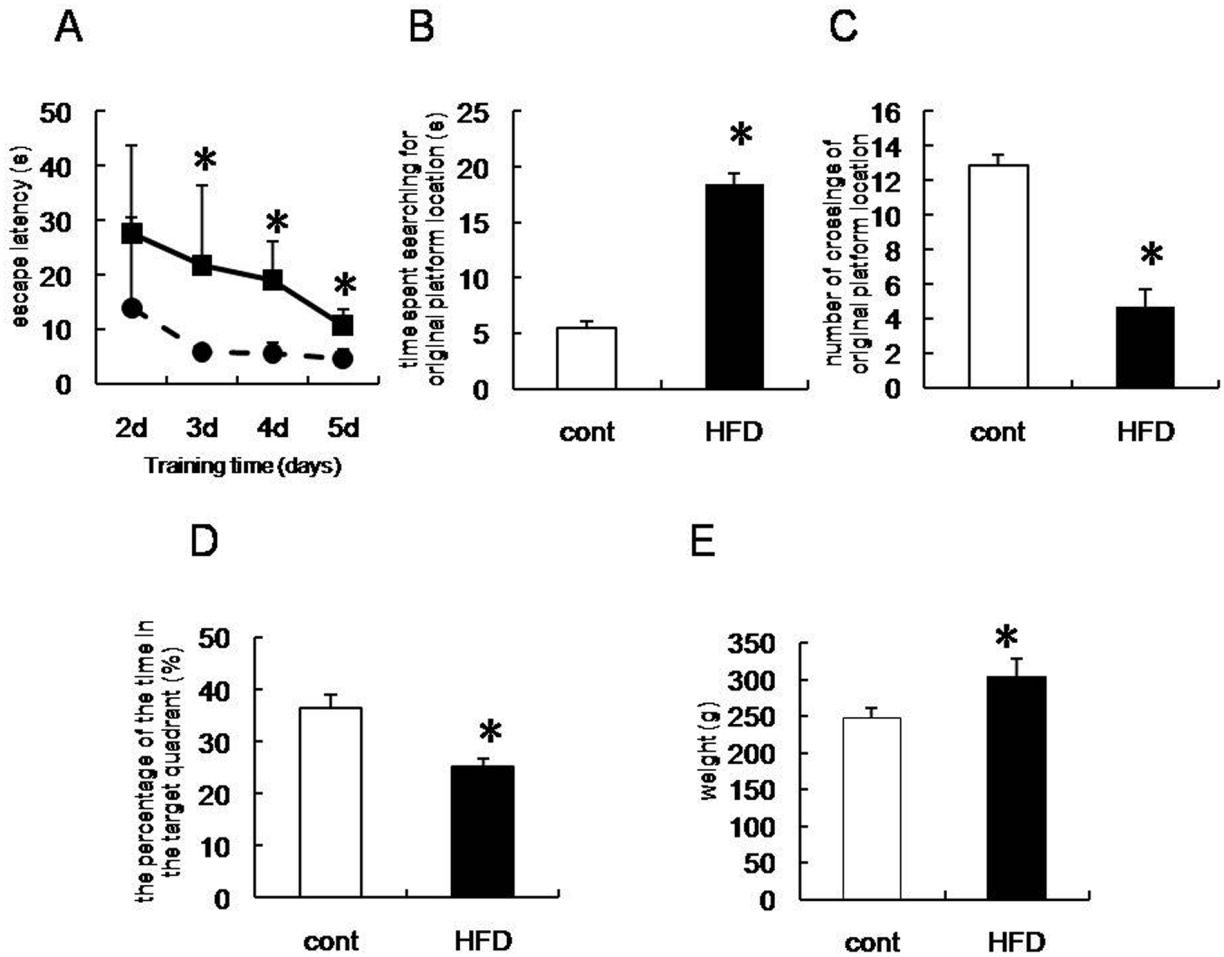
Cognitive performance assessed using the Morris water maze. (A) Learning performance of the animals was analyzed in the training trials by monitoring escape latency. (B) Memory retrieval performance was investigated in the probe trial (post-training) as the time required to search for the original platform location. (C) The number of times rats crossed over the location of the original platform. (D) The percentage of the time in the target quadrant. (E) Weight of control and high fat diet (HFD) rats after 6 weeks of being fed a normal or HFD, respectively. Control group (open bar): n = 8, HFD group (black bar): n = 8. Data are expressed as the mean ± SD; *p < 0.05 vs. control group.

Rats from each group were subjected to the Morris water maze to determine whether HFD affected cognitive impairment. The latency of the control group was short, and continued to decrease over the 4-day training period. In contrast, escape latency in the HFD group was significantly longer than the control group (days 3–5, Fig 1A). After removing the platform, HFD rats spent a longer time trying to find the location of the original platform than the control group (Fig 1B). Consistent with this, HFD rats crossed over the location of the original platform fewer times than rats in the control group (Fig 1C). In addition, there was a decline in the percentage time spent in the target quadrant in HFD rats compared with the control group (Fig 1D). These results suggest that HFD rats exhibited a decline in cognitive function. The body weight of HFD rats was significantly greater than the control group (Fig 1E).

### Increased ER stress in the hippocampus and frontal cortex of HFD rats

**Fig 2:**
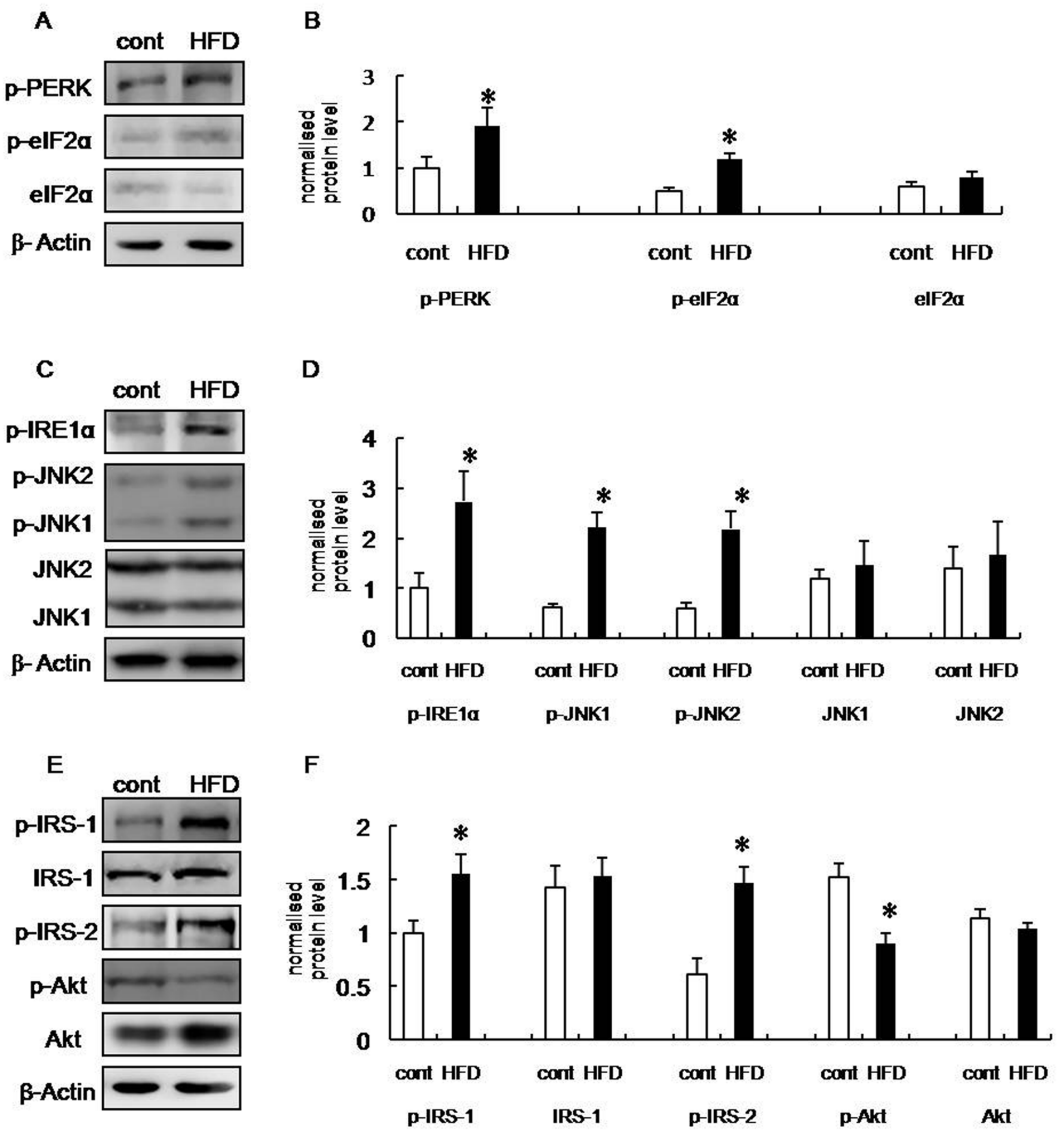
Increased ERS and insulin receptor signaling inhibition in hippocampus of HFD rats. (A) Western blot of p-PERK, p-eIF2α, and eIF2α in the hippocampus of control and HFD rats. (B) Statistical analysis of IOD of panel (A). (C) Western blot of p-IRE1α, p-JNK1, p-JNK2, JNK1, and JNK2 in the hippocampus of control and HFD rats. (D) Statistical analysis of IOD of panel (B). (E) Western blot of p-IRS-1, IRS-1, p-IRS-2, p-Akt, and Akt in the hippocampus of control and HFD rats. (F) Statistical analysis of IOD of panel (E). Control group (open bar): n = 5, HFD group (black bar): n = 5. Blots were digitized and the bands quantified using an image analysis system. Bars represent mean ± SD of five independent experiments. Data are expressed as the mean ± SD; *p < 0.05 vs. control group.

**Fig 3:**
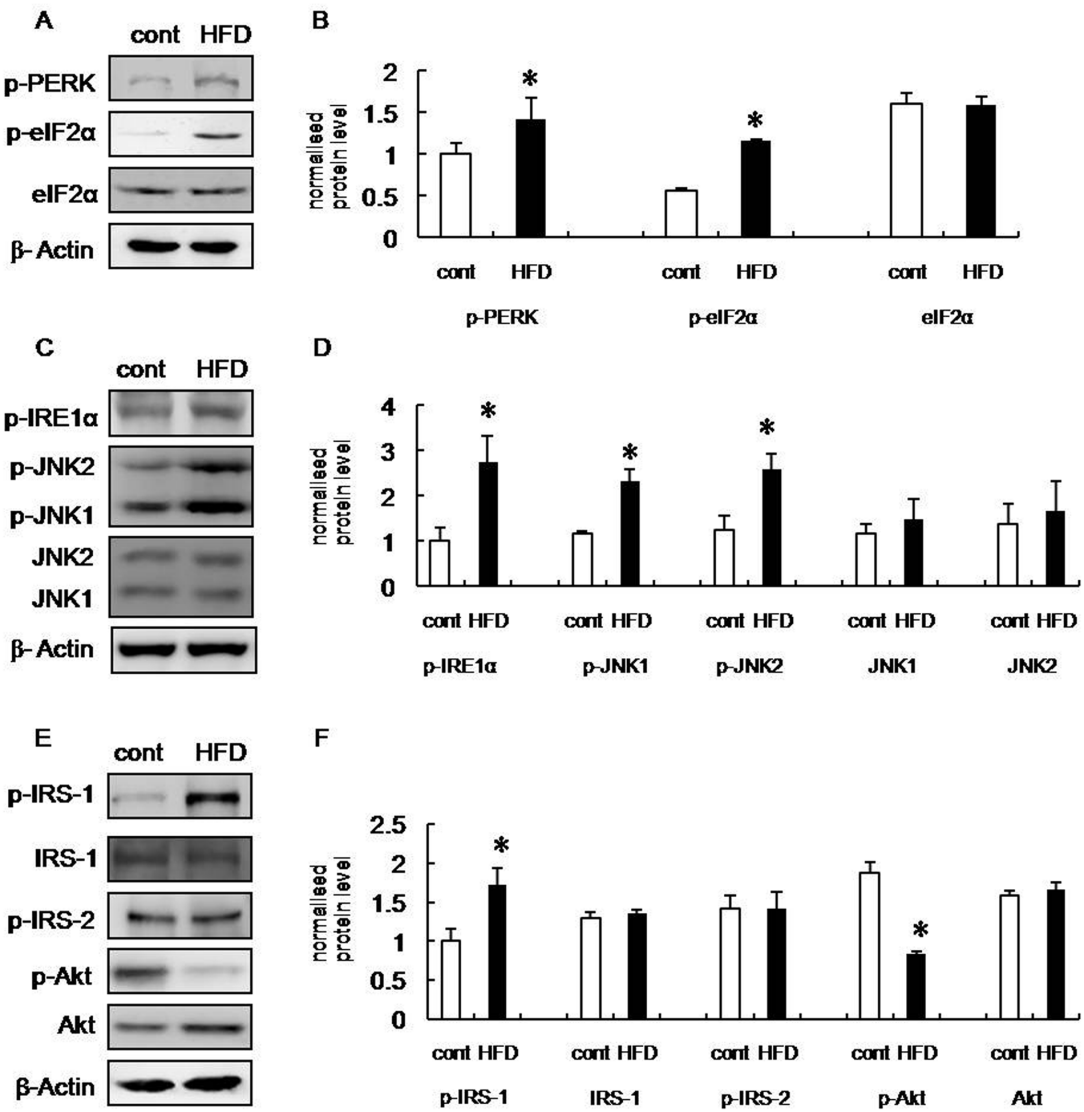
Increased ERS and insulin receptor signaling inhibition in cortex of HFD rats. (A) Western blot of p-PERK, p-eIF2α, and eIF2α in the frontal cortex of control and HFD rats. (B) Statistical analysis of IOD of panel (A). (C) Western blot of p-IRE1α, p-JNK1, p-JNK2, JNK1, and JNK2 in the frontal cortex of control and HFD rats. (D) Statistical analysis of IOD of panel (B). (E) Western blot of p-IRS-1, IRS-1, p-IRS-2, p-Akt, and Akt in the frontal cortex of control and HFD rats. (F) Statistical analysis of IOD of panel (E). Control group (open bar): n = 5, HFD group (black bar): n = 5. Blots were digitized and the bands quantified using an image analysis system. Bars represent mean ± SD of five independent experiments. Data are expressed as the mean ± SD; *p < 0.05 vs. control group.

To determine whether increased ER stress was associated with obesity, we assessed the expression patterns of several molecular markers of ER stress in the brains of HFD rats. PKR-like kinase (PERK) is an ER transmembrane protein kinase that phosphorylates the α subunit of translation initiation factor 2 (eIF2α) in response to ER stress. The phosphorylation status of PERK and eIF2α is therefore a key indicator of ER stress [16,17,18]. We analyzed the phosphorylation of PERK and eIF2α in hippocampus and frontal cortex extracts from HFD and control group rats. The phosphorylation of both PERK (Thr 980) and eIF2α (Ser 51) were increased significantly in brain extracts from HFD rats compared with control (Figs 2A, 2B, 3A and 3B), suggesting that the UPR had been activated.

Inositol-requiring kinase-1α (IRE-1α) is an additional indicator of ERS. During ERS, IRE-1α dissociates from 78-kD glucose-regulated/binding immunoglobulin protein (GRP78), which is an ER chaperone whose expression is increased during ERS, and subsequently undergoes autophosphorylation [19]. Therefore, we also assessed the phosphorylation of IRE-1α. Our data revealed that the phosphorylation of IRE-1α (Ser 724) was increased in the hippocampus and frontal cortex of HFD rats compared with control (Figs 2C, 2D, 3C and 3D). Taken together, these results suggest that cognitive impairment induced by obesity is associated with the induction of ERS.

Under ERS conditions, increased IRE-1α phosphorylation leads to the recruitment of tumor necrosis factor receptor-associated factor 2 (TRAF2), and the subsequent activation of JNK [20,21]. Therefore, we next assessed JNK phosphorylation (Thr 183/Tyr 185) and our data revealed that HFD rats exhibit increased JNK phosphorylation in the hippocampus and frontal cortex compared with control, suggesting that JNK was activated in these brain regions (Figs 2C, 2D, 3C and 3D).

### Inhibited insulin receptor signaling in the hippocampus and frontal cortex of HFD rats

Insulin receptor substrate-1 (IRS-1) and insulin receptor substrate-2 (IRS-2) are substrates for the insulin receptor tyrosine kinases. Serine phosphorylation of IRS-1, particularly by JNK, reduces insulin receptor signaling [22,23,24]. IRS-1 and -2 and PKB/Akt are important markers of insulin receptor signaling. Therefore, we examined the serine phosphorylation of these markers in the hippocampus and frontal cortex of HFD and control rats. Our data demonstrates that levels of phospho-IRS-1 (Ser 307) were increased in the hippocampus and frontal cortex, while IRS-2 serine phosphorylation (Ser 731) was increased only in the hippocampus of HFD rats. This suggests that insulin receptor signaling was inhibited in the brains of HFD rats (Figs 2E, 2F, 3E and 3F). In addition, insulin receptor signaling was impaired more significantly in the hippocampus, which was indicative of cognitive decline. Consistent with these observations, the activity of Akt, which is downstream of IRS-1 and -2, was also inhibited (Figs 2E, 2F, 3E and 3F). Collectively, these data supported the hypothesis that ERS was induced, and insulin receptor signaling impaired, in the hippocampus and frontal cortex of HFD rats.

### Endoplasmic reticulum stress inhibits insulin receptor signaling *in vitro*

**Fig 4:**
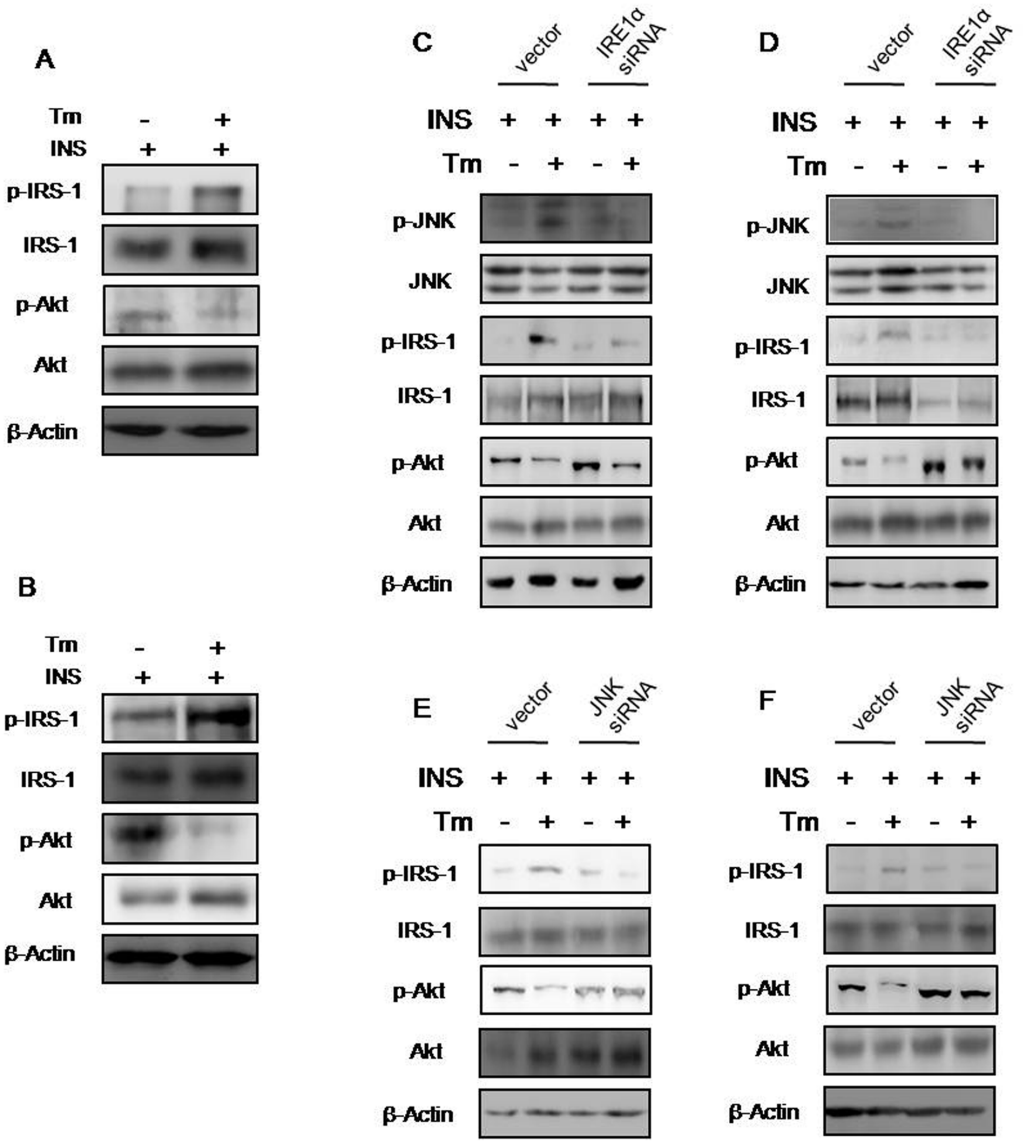
ERS inhibits insulin receptor signaling in primary rat hippocampal and cortical neurons. (A–B) Western blot analysis of the effect of 4 h tunicamycin (Tm) treatment on p-IRS-1, IRS-1, p-Akt, and Akt levels in primary rat hippocampal (A) and cortical neurons (B). (C–D) Western blot analysis of the effect of 4 h Tm treatment on p-JNK, JNK, p-IRS-1, IRS-1, p-Akt, and Akt levels in primary rat hippocampal (C) and cortical neurons (D) transfected with IRE1α siRNA. (E–F) Western blot analysis of the effect of 4 h Tm treatment on p-IRS-1, IRS-1, p-Akt, and Akt levels in primary rat hippocampal (E) and cortical neurons (F) transfected with JNK siRNA. Statistical analysis is shown in S1 Fig. Blots were digitized and bands quantified using an image analysis system.

To investigate whether ERS alters the effects of insulin, we treated hippocampal and cortical neurons with the ERS inducer tunicamycin (Tm). Treatment of cells with Tm for 4 h increased insulin-induced serine phosphorylation of IRS-1 (Ser 307) (Fig 4A and 4B, S1A and S1B Fig), while reducing Akt phosphorylation (Ser 473) (Fig 4A and 4B, S1A and S1B Fig). These findings suggest that experimental ERS inhibits insulin effects. Next, we examined the role of IRE1α and JNK in ERS-induced IRS-1 serine phosphorylation. IRE1α activity was inhibited by transfecting hippocampal and cortical neurons with IRE1α siRNA. This reversed ERS-induced JNK activity and serine phosphorylation of IRS-1 (Fig 4C and 4D, S1C and S1D Fig). To confirm these observations, JNK activity was inhibited by transfecting cells with JNK siRNA, which also inhibited ERS-induced IRS-1 serine phosphorylation (Fig 4E and 4F, S1E and S1F Fig). These results suggest that IRE1α promotes JNK-dependent serine phosphorylation of IRS-1, which inhibits insulin receptor signaling.

**Fig 5:**
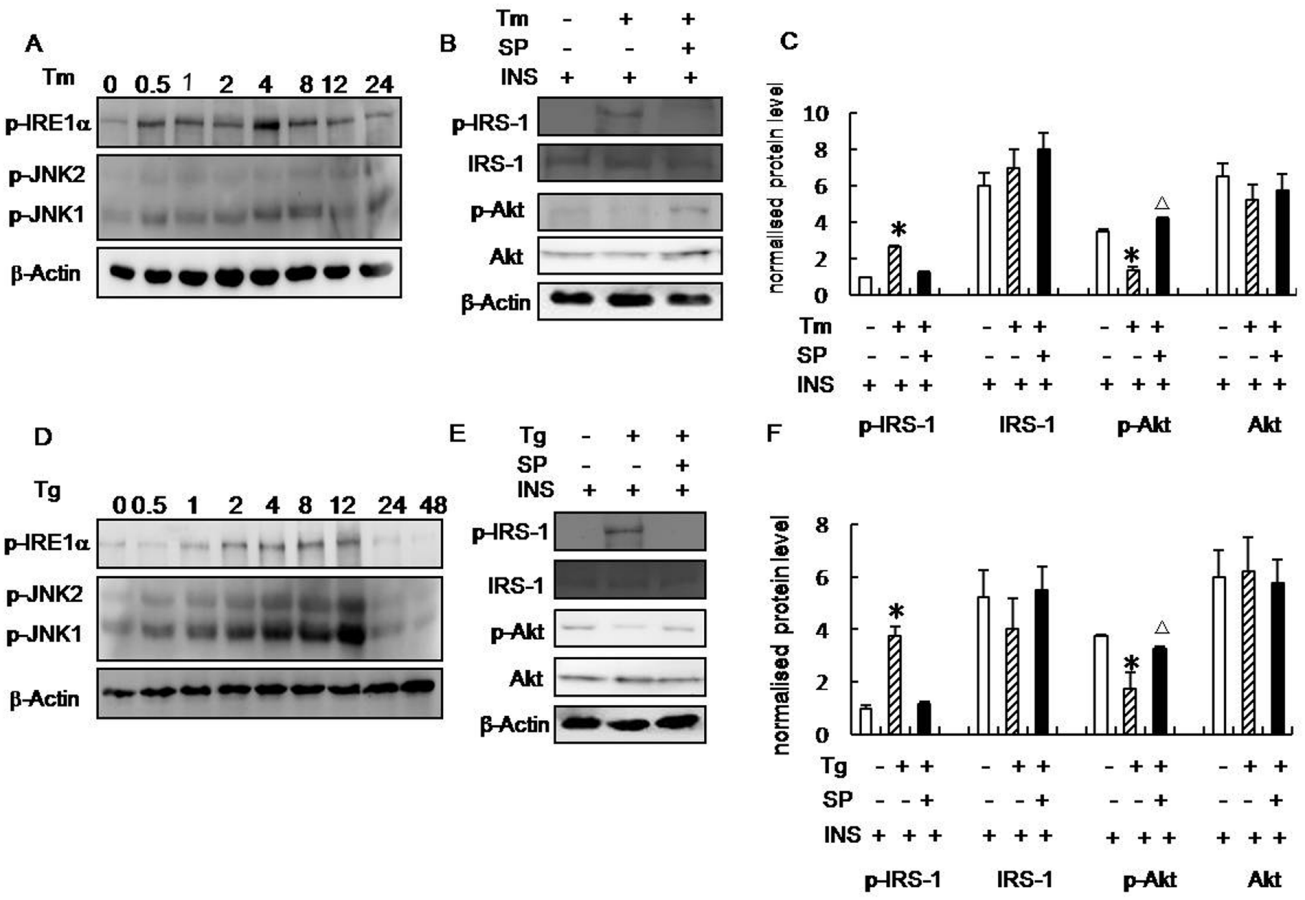
ERS inhibits insulin receptor signaling in SH-SY5Y cells. (A) Western blot analysis of p-IRE1α, p-JNK1, and p-JNK2 levels following treatment with tunicamycin (Tm) for 0, 0.5, 1, 2, 4, 8, 12, and 24 h. (B-C) Western blot analysis of p-IRS-1, IRS-1, p-Akt, and Akt levels following treatment with Tm for 4 h followed by treatment with SP600125 for 4 h. (D) Western blot analysis of p-IRE1α, p-JNK1, and p-JNK2 levels following treatment with thapsigargin (Tg) for 0, 0.5, 1, 2, 4, 8, 12, 24, and 48 h. (E-F) Western blot analysis of p-IRS-1, IRS-1, p-Akt, and Akt levels after treatment with Tg for 12 h followed by treatment with SP600125 for 4 h. Tm(or Tg)-/SP-/INS+ (open bar): n = 4, Tm(or Tg)+/SP-/INS+ (bias bar): n = 4, Tm(or Tg)+/SP+/INS+ (black bar): n = 4. Blots were digitized and the bands were quantified using an image analysis system. Bars represent the mean ± SD from four independent experiments. Data are expressed as the mean ± SD; *p < 0.05 vs. Tm(or Tg)-/SP-/INS+. ^△^ *p* < 0.05 vs. Tm(or Tg)+/SP-/INS+.

In addition, we treated SH-SY5Y cells with tunicamycin (5 μg/ml) or thapsigargin (3 μg/ml), which are both commonly used to induce ERS. Treatment with Tm for 4 h or thapsigargin (Tg) for 12 h significantly increased insulin-induced phosphorylation of IRE1α and JNK (Fig 5A and 5D). Next, we examined the relationship between ERS and insulin receptor signaling in SH-SY5Y cells. Tm and Tg significantly increased the serine phosphorylation of IRS-1 (Fig 5B, 5C, 5E and 5F) and reduced Akt phosphorylation (Fig 5B, 5C, 5E and 5F). These findings suggest that experimental ERS inhibits the effects of insulin. We then examined the role of JNK in ERS-induced IRS-1 serine phosphorylation. Inhibiting JNK activity using the chemical inhibitor SP600125 reversed ERS-induced IRS-1 serine phosphorylation (Fig 5B, 5C, 5E and 5F). These results suggested that ERS promoted JNK-dependent serine phosphorylation of IRS-1, which in turn inhibited insulin receptor signaling.

## Discussion

Despite an increase in research aimed at investigating the relationship between obesity and cognitive impairment, the pathogenesis underlying this disease remains unclear. ERS can be induced by aberrant changes in various intracellular processes, such as protein folding/modification, redox balance, energy consumption, and Ca^2+^ regulation. A pathogenic role for chronic ERS has been identified in neurodegenerative diseases that are characterized by excessive intracellular accumulation and aggregation of misfolded proteins, for example in AD, Parkinson’s disease, Huntington’s disease, and amyotrophic lateral sclerosis (ALS) [25,26,27]. In the current study, we found that the phosphorylation of both PERK and eIF2α, which are important ERS indicators, were significantly increased in hippocampus and frontal cortex of HFD rats, suggesting the presence of increased ERS in the hippocampus and frontal cortex of HFD rats.

Several studies in liver and adipose tissue have revealed that nutrient excess-induced chronic ER stress plays a role in the pathogenesis of diabetes, and specifically contributes to hepatic steatosis [28], beta cell loss, and insulin resistance [14,29,30]. IRS-1, a docking protein downstream of the insulin receptor, has been shown to be a target of prolonged ER stress via inhibition by the IRE-1/c-Jun NH2-terminal kinase (JNK) pathway in the liver of obese mice [14]. However, the relationships among IRE1, JNK and insulin receptor signaling had not been previously verified in brains of obese rats. In this study, we demonstrated that phosphorylation of IRE1α, which is an ERS sensor, JNK1 and JNK2 was increased in the hippocampus and frontal cortex of HFD rats. These results also suggest that ERS is increased in the hippocampus and frontal cortex of obese rats, which is consistent with the observed increases in phosphorylation of PERK and eIF2α.

Tissue responses to insulin require the IRS-1 and IRS-2 proteins [31], which are tyrosine phosphorylated by the insulin receptor (IR), thereby recruiting phosphatidylinositol 3’ kinase (PI3K), which in turn stimulates the activation of Akt by Pdk1 and initiates diverse signaling pathways. In contrast, serine and threonine (Ser/Thr) phosphorylation of IRS-1 by multiple kinases in response to inflammatory cytokines, fatty acids, and insulin itself impaired insulin receptor signaling in cultured cells [32]. Of the more than 200 Ser/Thr residues in IRS-1, approximately 30 are known to be phosphorylated in insulin-treated cells or human muscle [33,34]. The Ser 307, Ser 636/639, Ser 616 sites on IRS-1 have been shown to be involved in insulin receptor signaling in obesity and AD [35,36]. An IRS-1 Ser 307 mutation was shown to protect against tumor necrosis factor alpha induced inhibition of IRS-1 tyrosine (Tyr) phosphorylation via the JNK kinase pathway [23]; thus, Ser 307 phosphorylation is frequently cited as a cause of stress-induced insulin resistance. Ob/ob mice lacking JNK1 exhibit reduced Ser 307 phosphorylation and improved insulin sensitivity, with decreased circulating insulin concentrations [37]. In this study, increased IRS-1 Ser 307 phosphorylation and decreased Akt phosphorylation, both indictors of impaired insulin receptor signaling, were observed in the hippocampus and frontal cortex of HFD rats, although a recent study demonstrated that IRS-1 Ser 307 is a positive regulatory site that sustains peripheral insulin signaling and moderates the severity of insulin resistance in mice [38]. We used siRNA for IRE1α and JNK in primary hippocampal and cortical neurons, treated with Tm and insulin, to demonstrate that IRE1α-dependent JNK activation mediates the induction of ERS and the suppression of insulin receptor signaling, which is characterized by increased IRS-1 Ser 307 phosphorylation and decreased AKT phosphorylation. These findings suggest that ERS and impaired insulin receptor signaling in the brain could contribute to the development of cognitive impairment induced by HFD.

Several reports have indicated that IRS-2 participates in reducing amyloid deposition and cognitive deficits in a transgenic mouse model of AD [39]. Furthermore, IRS-2 signaling impairs dendritic spine formation [40]. In our study, IRS-2 Ser 731 phosphorylation increased in the hippocampus of HFD rats but not the frontal cortex, indicating IRS-2 signaling is impaired in hippocampus under high fat diet conditions. However, it remains unclear whether IRS-2 Ser 731 phosphorylation was activated by JNK. The beneficial effects of IRS-2 deletion on AD pathology parallel its effects on lifespan, with less IRS-2 signaling resulting in extended lifespan in mice [41]. Therefore, there is an apparent dichotomy between the neuroprotective effects of insulin signaling in the CNS and its deleterious actions on lifespan and memory.

In the current study, we selected a HFD-treated rat model that is commonly used in pharmacological studies of obesity [42,43]. In the Morris water maze test, HFD rats exhibited severe cognitive deficits, which are consistent with previous findings [44].

There are several limitations to the present study. First, the relationship between ERS and insulin receptor signaling was only verified *in vitro*. Second, although IRS-1 Ser 307 phosphorylation plays a role in JNK-induced impaired insulin signaling, other serine phosphorylation sites were not examined. It is possible that JNK impairs insulin receptor signaling at multiple serine phosphorylation sites, which eventually promotes cognitive impairment induced by HFD. In addition, the relationship between ERS and insulin receptor signaling is very complex, so whether or not insulin receptor signaling affects ERS in a feedback loop needs to be studied further.

In summary, we describe a novel relationship between ERS and insulin receptor signaling. Our data suggest that interventions that regulate the ERS response and insulin receptor signaling in the brain may provide novel opportunities for treating cognitive impairment induced by obesity. However, further studies are required to determine whether structural changes or other variations in the ER could affect insulin secretion or insulin effects, and whether abnormal endoplasmic reticulum function in the CNS could modulate insulin effectiveness in peripheral tissues.

## Supporting Information

S1 Fig Quantification of the results shown in Fig 4. (A–B) Statistical analysis of A–B of Fig 4. IOD of p-IRS-1, IRS-1, p-Akt, and Akt in primary rat hippocampal (A) and cortex neurons (B) after treatment with tunicamycin (Tm) for 4 h. (C–D) Statistical analysis of C–D of Fig 4. IOD of p-JNK, JNK, p-IRS-1, IRS-1, p-Akt, and Akt in primary rat hippocampal (C) and cortex neurons (D) transfected with IRE1α siRNA after treatment with Tm for 4 h. (E–F) Statistical analysis of (C–D) of Fig 4. IOD of p-IRS-1, IRS-1, p-Akt, and Akt in primary rat hippocampal (E) and cortex neurons (F) transfected with JNK siRNA after treatment with Tm for 4 h. Blots were digitized and bands quantified using an image analysis system. Data are expressed as the mean ± SD of four independent experiments; *p < 0.05 vs. Tm-/INS+ with or without vector transfection. ^△^ *p* < 0.05 vs. Tm+/INS+ with vector transfection.
(TIF)
