# Supplementary figures and images for: Endoplasmic Reticulum Stress Impairs Insulin Receptor Signaling in the Brains of Obese Rats
Source: PLoS One. 2015 May 15;10(5):e0126384. doi: 10.1371/journal.pone.0126384 (PMC4433117; doi:10.1371/journal.pone.0126384)

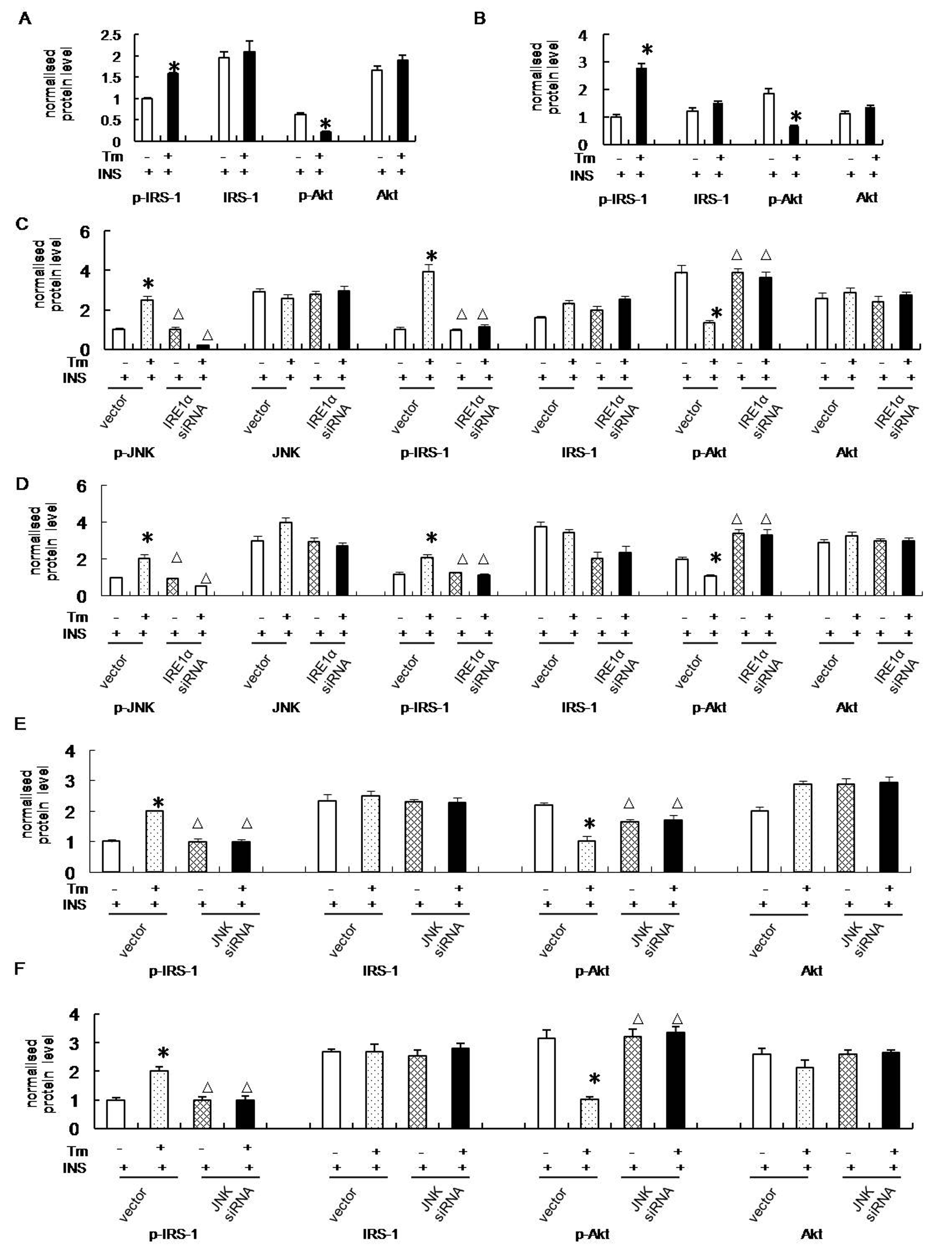

Supplement: S1 Fig — (A–B) Statistical analysis of A–B of Fig 4. IOD of p-IRS-1, IRS-1, p-Akt, and Akt in primary rat hippocampal (A) and cortex neurons (B) after treatment with tunicamycin (Tm) for 4 h. (C–D) Statistical analysis of C–D of Fig 4. IOD of p-JNK, JNK, p-IRS-1, IRS-1, p-Akt, and Akt in primary rat hippocampal (C) and cortex neurons (D) transfected with IRE1α siRNA after treatment with Tm for 4 h. (E–F) Statistical analysis of (C–D) of Fig 4. IOD of p-IRS-1, IRS-1, p-Akt, and Akt in primary rat hippocampal (E) and cortex neurons (F) transfected with JNK siRNA after treatment with Tm for 4 h. Blots were digitized and bands quantified using an image analysis system. Data are expressed as the mean ± SD of four independent experiments; *p < 0.05 vs. Tm-/INS+ with or without vector transfection. △ p < 0.05 vs. Tm+/INS+ with vector transfection. (TIF) [file pone.0126384.s001.tif]
